# Comparative study of human mitochondrial proteome reveals extensive protein subcellular relocalization after gene duplications

**DOI:** 10.1186/1471-2148-9-275

**Published:** 2009-11-30

**Authors:** Xiujuan Wang, Yong Huang, Dennis V Lavrov, Xun Gu

**Affiliations:** 1 Interdepartmental Genetics Program, Iowa State University, Ames, IA 50011, USA; 2 Bioinformatics and Computational Biology Program, Iowa State University, Ames, IA 50011, USA; 3 Department of Ecology, Evolution and Organismal Biology, Iowa State University, Ames, IA 50011, USA; 4 Department of Genetics, Development and Cell Biology, Iowa State University, Ames, IA 50011, USA

## Abstract

**Background:**

Gene and genome duplication is the principle creative force in evolution. Recently, protein subcellular relocalization, or neolocalization was proposed as one of the mechanisms responsible for the retention of duplicated genes. This hypothesis received support from the analysis of yeast genomes, but has not been tested thoroughly on animal genomes. In order to evaluate the importance of subcellular relocalizations for retention of duplicated genes in animal genomes, we systematically analyzed nuclear encoded mitochondrial proteins in the human genome by reconstructing phylogenies of mitochondrial multigene families.

**Results:**

The 456 human mitochondrial proteins selected for this study were clustered into 305 gene families including 92 multigene families. Among the multigene families, 59 (64%) consisted of both mitochondrial and cytosolic (non-mitochondrial) proteins (mt-cy families) while the remaining 33 (36%) were composed of mitochondrial proteins (mt-mt families). Phylogenetic analyses of mt-cy families revealed three different scenarios of their neolocalization following gene duplication: 1) relocalization from mitochondria to cytosol, 2) from cytosol to mitochondria and 3) multiple subcellular relocalizations. The neolocalizations were most commonly enabled by the gain or loss of N-terminal mitochondrial targeting signals. The majority of detected subcellular relocalization events occurred early in animal evolution, preceding the evolution of tetrapods. Mt-mt protein families showed a somewhat different pattern, where gene duplication occurred more evenly in time. However, for both types of protein families, most duplication events appear to roughly coincide with two rounds of genome duplications early in vertebrate evolution. Finally, we evaluated the effects of inaccurate and incomplete annotation of mitochondrial proteins and found that our conclusion of the importance of subcellular relocalization after gene duplication on the genomic scale was robust to potential gene misannotation.

**Conclusion:**

Our results suggest that protein subcellular relocalization is an important mechanism for the retention and gain of function of duplicated genes in animal genome evolution.

## Background

Gene duplication is an important evolutionary process that plays a key role in generating new genomic information in all the three domains of life: Eubacteria, Archaea and Eukarya [1-5]. Various processes can cause gene duplication on the molecular level, including unequal crossovers, retroposition, or whole chromosome/genome duplication [6-8]. New functional genes resulting from gene duplication are retained in the genome through the processes of subfunctionalization and neofunctionalization [9,10]. Subfunctionalization refers to a situation when each of the daughter genes adopts only a partial function of the parental gene, while neofunctionalization refers to the gain of new functions by the duplicate, usually related to their ancestor's function [4].

From the evolutionary perspective, neofunctionalization presents more interest than subfunctionalization because it results in the increase of the total genetic information [11]. Several mechanisms have been invoked to explain the gain of novel gene function for duplicated genes such as dosage compensation, epigenetic complementation, moonlighting, and catalytic promiscuity [12-17]. Interestingly, functional divergence can in some cases precede (and facilitate) gene duplication through allelic divergence [18]. Recently, protein subcellular relocalization or neolocalization has been proposed as a key event for generating new functional genes after duplication [19,20]. Such neolocalization can be achieved by the gain or loss of N-terminal targeting peptide sequences that can direct the products of duplicated genes from the cytosol to mitochondria, endoplasmic reticulum and chloroplast or vice versa.

The idea of subcellular relocalization underlying the gain of function for duplicated genes has been tested in two yeast genomes by Marques et al. (2008). The authors demonstrated that about one-third of the duplicated genes retained in the yeast genomes had undergone protein subcellular relocalization following whole genome duplication [20]. A few anecdotal observations suggest that neolocalization after duplication also occurs in animal genomes [21,22], however the magnitude of this process has not been explored. In this study, we performed a systematic survey of subcellular relocalization following gene duplication in the human genome by analyzing nuclear-encoded mitochondrial protein families.

Mitochondria, cell organelles present in nearly all eukaryotes, are instrumental for the production of ATP through oxidative phosphorylation process, and are also involved in heme biosynthesis, cell metabolism, apoptosis, and Fe/S cluster biosynthesis. The complex functions of mitochondria demand a proteome composed of over a thousand of proteins, more than 98% of which are nuclear encoded, which suggests these organelles should play a major role in the process of neolocalization [23]. Hence, the exploration of nuclear encoded mitochondrial gene families is an ideal system to test subcellular relocalization of duplicated genes in the evolution of animal genomes.

## Results and Discussion

### Subcellular relocalization as a mechanism underlying protein functional divergence

For this study we retrieved 456 human mitochondrial proteins from MitoP2 database http://www.mitop.de:8080/mitop2/ that were also annotated as mitochondrial proteins in Swissprot database. Reciprocal blasting and single linkage clustering were carried out to group the proteins into 305 families, among which 195 were single gene families (not considered here) and 110 were multigene families. After removing 18 families with members that appeared to be alternative splicing products or annotation artifacts, we obtained the final dataset of 92 multigene families for further analysis (Table 1). These 92 families can be classified into two categories based on their designated subcellular localizations: 1) mitochondrial-cytosolic (mt-cy) families that consist of at least one protein member localized in mitochondria and at least one in another (non-mitochondrial) cellular compartment, and 2) mitochondrial only (mt-mt) families that are composed of protein members localized exclusively in mitochondria. The mt-cy category contained 59 families with 144 mitochondrial proteins and 196 non-mitochondrial proteins in humans, while the mt-mt category had 33 families with 79 human mitochondrial proteins. This result suggests that around two thirds of the mitochondrial multigene families have undergone subcellular relocalization after duplication.

**Table 1:** Summary of human mitochondrial multigene families

			Total number of proteins		Phylogenetic interval		
				
Category	Multigene families^a^	Total number of family	mitochondrial	non-mitochondrial	Before vertebrates	At the root of vertebrates	After the emergence of fish
Mitochondrial- cytosolic families (mt-cy families)	ACAA2, ALDH1B1, CPT2, GPAM, HMGCS2, NDUFA4, SH3BP5, HIBADH, ABCB10, CABC1, ARG2, ATP5A1, BNIP3, CYP11B2, CA5A, CPS1, CDS2, CYB5B, DECR1, DGUOK, DLD, DNAJA3, GFM1, TUFM, FTMT, GK, SHMT2, GPX4, HSPA9, ALAS2, HTRA2, HK1, IDH2, MTIF2, PPA2, AK3, CKMT2, ME2, MFN2, MGST1, MIPEP, NME4, NFS1, OAS2, OPA1, SLC25A15, PCK2, PHB, PPIF, PRDX3, SIRT3, IARS2, SARS2, DNAJC19, TMLHE, TOP1MT, TRAP1, TST, OXR1	59	144	196	27^b^ (45.8%)	29^b ^(49.2%)	3^b^(5%)

Mitochondrial- mitochondrial families (mt-mt families)	AIFM1, MAOA, ATP5G2, BCL2L1, D2HGDH, GLUD2, GRPEL1, LETM1, MCART1, PMPCB, MTCH2, MTERF, ENDOG, OAT, BCKDHA, PDHB, PDK4, PDP2, MTRF1, RHOT1, SCO2, TIMM17A, TOMM40, VDAC3, COX4I2, COX7A1, COX6A1, COX6B1, MCCC2, OXCT2, MRPS18A, MRPS10, MRPS30	33	79	-	8 (24%)	19 (57.6%)	6 (18.4%)

^a ^Families are listed using gene names of mitochondrial members.

^b ^For families that underwent multiple rounds of duplications, only the duplications followed by subcellular relocalizations were considered.

For each human protein in the 92 gene families, we retrieved orthologs in mouse (*Mus musculus*), chicken (*Gallus gallus*), fish (*Danio rerio*), fruit fly (*Drosophila melanogaster*), mosquito (*Anopheles gambiae*) and nematode (*Caenorhabditis elegans*) from the Homologene database at NCBI. Phylogenetic analyses were conducted for each family and the time of occurrence of gene duplications in relationship to major divergences in animal evolution was evaluated. Because this study is based on the human mitochondrial proteome, only branches of the phylogenetic tree leading to humans were investigated. For mitochondrial-cytosolic (mt-cy) families that underwent several rounds of duplications, we only considered the duplications that were followed by subcellular relocalizations.

Among the 59 mt-cy families, twenty-seven (45.8%) were inferred to undergo gene duplication prior to the protostome/deuterostome divergence, twenty-nine (49.2%) after the protostome/deuterostome divergence but before that of fish/tetrapods and only three families (5%) within the tetrapod lineage (Table 1). Among the 33 mt-mt families, eight (24%) underwent gene duplication prior to the protostome/deuterostome split, nineteen (57.6%) prior to the fish/tetrapod split and six (18.4%) within the tetrapod lineage. The observation that the majority of investigated families experienced gene duplication between the protostome/deuterostome and fish/tetrapod divergences correlates well with the two rounds of genome duplication at the early stage of vertebrate evolution [24,25]. However, the scarcity of more recent subcellular relocalization events is surprising, especially considering very high rates of gene birth and death in animal genomes [26].

### Bidirectional relocalization of proteins encoded by duplicated genes in mitochondrial-cytosolic (mt-cy) two-gene families

In order to get insight into the direction of protein subcellular relocalization, we explored mt-cy gene families in which human genes are represented by two copies, one functioning in the cytosol and the other in mitochondria. Among the 24 such families, one third appeared to have its original function in mitochondria with the products of duplicated genes being relocalized to other cellular compartments, another third showed the opposite direction of protein relocalization and for the rest the direction of relocalization could not be determined due to the lack of outgroup information.

**Figure 1:**
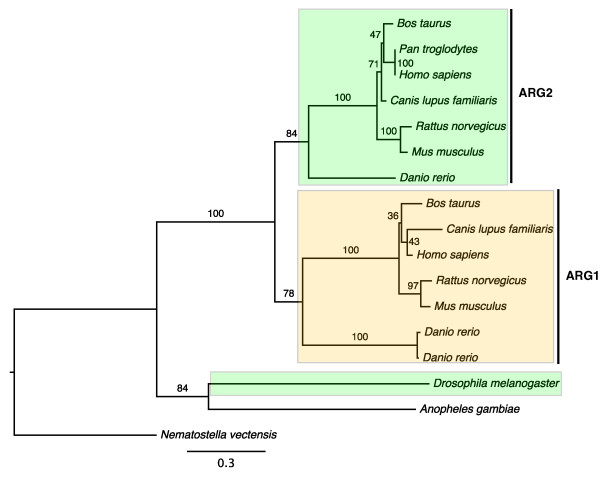
**Maximum likelihood phylogeny of the arginase family**. Numbers indicate bootstrap support based on 100 replicates. ARG1: Type I arginases; ARG2: Type II arginases. Colored boxes indicate annotated and/or predicted subcellular locations of the proteins: cytoplasm (yellow) and mitochondria (green). There is no subcellular information for the proteins in *Anopheles gambiae *and *Nematostella vectensis*.

The arginase family, encoding enzymes that catalyze the hydrolysis of arginine to ornithine and urea, is an example of neolocalization to the cytosol. Phylogenetic analysis of this family shows that the product of the ancestral gene had an original localization in mitochondria. Following a gene duplication in the lineage leading to vertebrates, type I arginase (ARG1) has relocalized to the cytoplasm while type II arginase (ARG2) retained its ancestral mitochondrial location (Figure 1). The evolutionary rates remain similar after the divergence of ARG1 and ARG2 genes. Sequence comparisons indicated that N-terminal mitochondrial targeting signals were not found in ARG1 in either human or mouse, but are present in all ARG2 sequences. The loss of N-terminal mitochondrial signals suggests that ARG1 could not be transported into mitochondria and is retained in the cytoplasm.

**Figure 2:**
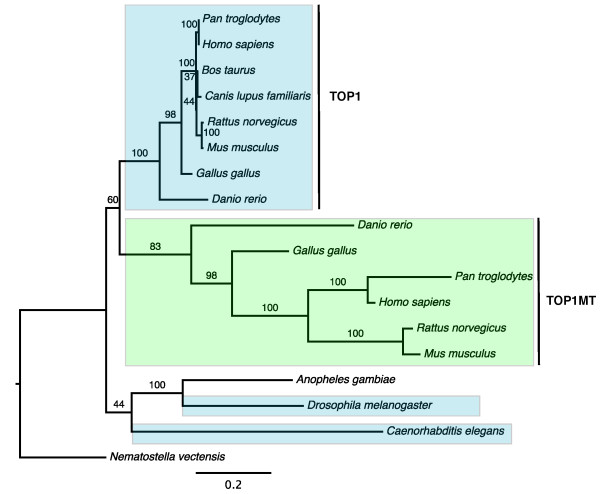
**Maximum likelihood phylogeny of the DNA topoisomerase typeIB family**. Numbers indicate bootstrap support based on 100 replicates. TOP1: DNA topoisomerase 1; TOP1MT: mitochondrial DNA topoisomerase 1. Colored boxes indicate annotated and/or predicted subcellular locations of the proteins: nucleus/cytoplasm (blue) and mitochondria (green). There is no subcellular information for the proteins in *Anopheles gambiae *and *Nematostella vectensis*.

The type IB subfamily of DNA topoisomerases that includes DNA topoisomerase 1 (TOP1) and mitochondrial DNA topoisomerase 1 (TOP1MT) presents an example of neolocalization to mitochondria. DNA topoisomerases control DNA topological states by catalyzing the transient breaking and rejoining of single strand DNA, allowing DNA strands or double helices to pass through each other [27]. These enzymes are essential in maintaining DNA topology during replication, transcription, recombination and DNA repair. Our phylogenetic analysis of the type IB subfamily revealed a gene duplication that occurred early in vertebrate evolution. Following this duplication, the product of one copy of the gene, TOP1MT, relocalized to mitochondria while the product of another - TOP1, retained its ancestral cytoplasmic and nuclear locations (Figure 2).

Both TOP1 and TOP1MT consist of four domains: a N-terminal localization domain, a core domain, a linker domain and a C-terminal domain [28]. Sequence comparisons show that the N-terminal domain of TOP1MT consists of a mitochondrial targeting signal, while the N-terminus of TOP1 contains a nuclear localization signal. This implies that the change in the N-terminal targeting sequence of TOP1MT helped the protein direct itself to mitochondria and eventually to acquire a new mitochondrial function. It should be noted that the lack of TOP1MT in invertebrates does not mean that type I topoisomerases are not needed for mitochondrial replication and transcription in this group. Recent studies have shown that DNA topoisomerase IIIα from the type IA subfamily has mitochondrial localization in *Drosophila melanogaster *[29,30].

**Figure 3:**
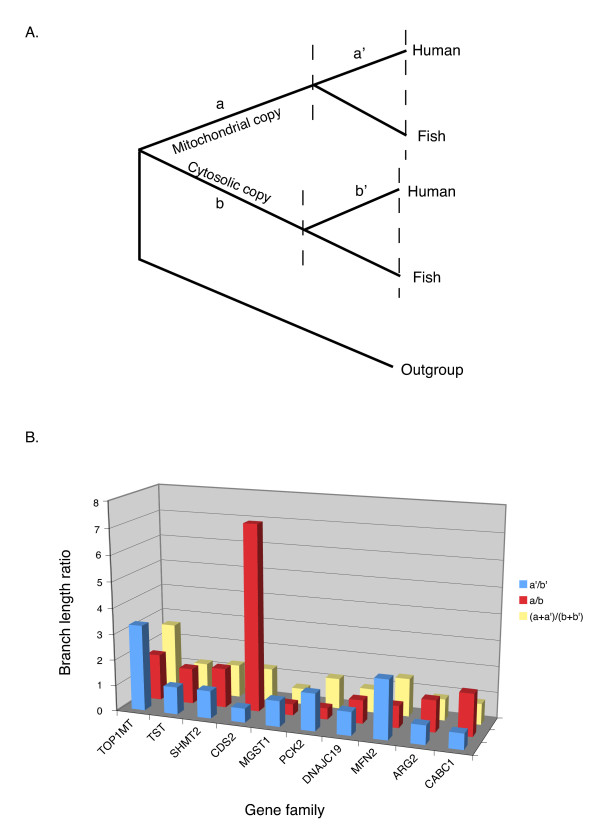
**Evolutionary rates in mitochondrial vs. non-mitochondrial proteins**. (A) A schematic phylogeny of a mt-cy two gene family with gene duplication occurred in the vertebrate lineage. Branch lengths before the divergence between fish and tetrapods are marked as a and b for mitochondrial and cytosolic proteins, respectively. The corresponding average branch lengths after this divergence are marked as a' and b'. (B) The ratios of branch lengths for mitochondrial vs. nuclear paralogs (a/b, a'/b', and (a+a')/(b+b')) were calculated on the maximum likelihood topologies as illustrated in (A) with the exception of the TST family, for which the divergence between birds (chicken) and mammals was used. TOP1MT, TST, SHMT2 and CDS2 families had undergone relocalization from cytosol to mitochondria, while the remaining 6 families had the opposite direction of relocalizations.

Does subcellular relocalization following duplication influence protein evolutionary rates? To answer this question we analyzed the relative evolutionary rates of duplicated genes in the mt-cy two gene families with well-resolved phylogenies and outgroup data. Three hypotheses were investigated: 1) Mitochondrial proteins generally have higher evolutionary rates comparing to their cytosolic counterparts; 2) The proteins involved in neolocalization have higher evolutionary rates; 3) Proteins undergo faster evolution following duplication/neolocalization due to functional relaxation or positive selection, with the evolutionary rates decreasing over time. To test the first two hypotheses, we compared the average branch lengths leading to mitochondrial and nuclear paralogs following a gene duplication [(a+a')/(b+b') in Figure 3A]. For the third hypothesis, we compared branch length ratio of mitochondrial vs. cytosolic proteins before and after the divergence between tetrapods and fish (a/b and a'/b' in Figure 3A). None of the proposed hypotheses was supported by our data (Figure 3B). Although some families had clearly uneven rates of evolution in mitochondrial vs. cytosolic proteins (e.g., Figure 2), most of the families displayed overall branch length ratios close to 1 regardless the direction of relocalization (Figure 3B). Similarly in some families mitochondrial proteins had higher evolutionary rates earlier in evolution (a/b>1) but lower rates at later stages (a'/b'<1) while in others an opposite pattern was observed (Figure 3B).

### Multiple subcellular relocalizations after gene duplications

**Figure 4:**
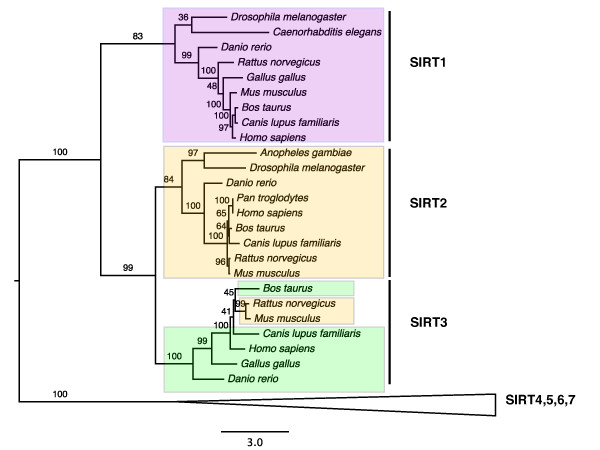
**Maximum likelihood phylogeny of the class I sirtuin family**. Numbers indicate bootstrap support based on 100 replicates. SIRT1: Sirtuin 1; SIRT2: Sirtuin 2; SIRT3: Sirtuin 3. Colored boxes indicate annotated and/or predicted subcellular locations of the proteins: nucleus (purple), cytoplasm (yellow) and mitochondria (green). SIRT3 in *Rattus norvegicus *and *Mus musculus *lost the mitochondrial N-terminal targeting signal and thus were retained in the cytoplasm.

In addition to the two-gene families discussed above, there are 35 mt-cy families with three or more (8 on average) members. Based on the current cellular component annotation in Swissprot database, we inferred that at least one third of these families had undergone multiple subcellular relocalizations. Class I sirtuin family presents a relatively simple example. In humans this family consists of SIRT1, SIRT2 and SIRT3 that regulate transcriptional repression [31]. SIRT1, located in the nucleus, is a deacetylase that regulates the tumor suppressor p53, NF-κB signaling, and FOXO transcription factors. SIRT2 is a cytoplasmic protein that deacetylates Lys40 of α-tubulin. Finally, SIRT3 is localized to the mitochondrial matrix [32,33]. The phylogeny of class I sirtuins suggests that the first round of duplication generated two copies with one copy (SIRT1) localized to the nucleus, while the other copy duplicated again resulting in one cytoplasmic copy (SIRT2) and one mitochondrial copy (SIRT3) (Figure 4). The N-terminal sequence analysis indicated the mitochondrial targeting signal was present in SIRT3 in all vertebrates on the tree except *Mus musculus *and *Rattus norvegicus*. The loss of the N-terminal mitochondrial targeting signal of SIRT3 in rodents suggests the loss of relocalization to mitochondria, an inference supported by an experimental demonstration that mouse SIRT3 actually locates to cytoplasm [34].

### Expansion of mitochondrial proteome by gene duplications

The presence of 33 mt-mt families among the 92 multigene families supports the notion that gene duplication also contributes to mitochondrial proteome expansion [35], although the average family size of mt-mt families is smaller than that of mt-cy families (2.4 vs 5.7). Our phylogenetic analyses of these mt-mt families showed that such duplications occurred at different stages in animal evolution, predating the divergence of the protostome/deuterostome lineages, within the vertebrate lineage, and within the mammalian lineage. In general, these families consist of proteins with similar functions that have been retained by subfunctionalization as different subunits or isoforms. Furthermore, the expression of these genes often shows tissue specificity such that one copy in the gene family is expressed ubiquitously, while the other(s) is/are expressed in specific tissues. For example, human SCO2 homolog (SCO cytochrome oxidase deficient homolog 2 (yeast)) is expressed ubiquitously while the SCO1 homolog is predominantly expressed in muscle, heart, and brain, the tissues featured by high rates of oxidative phosphorylation [36].

### Evolutionary modifications of relocalized proteins at sequence level

**Figure 5:**
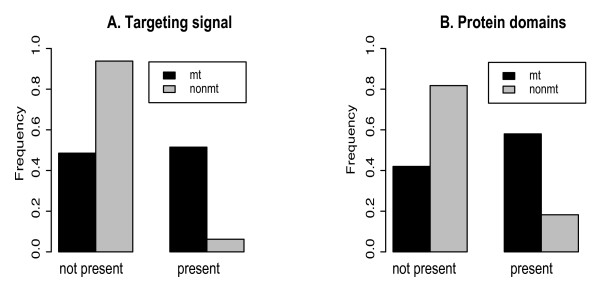
**The presence of mitochondrial N-terminal targeting signals (A) and mitochondrial Pfam domains (B) for human mitochondrial (mt) and non-mitochondrial (nonmt) proteins**. N-terminal mitochondrial targeting signals were inferred for proteins in mt-cy families based on targetP predictions [38]. Mitochondrial Pfam domains refer to those domains that were found only in eukaryotic (excluding human) mitochondrial proteins [40].

Proteins synthesized in the cytosol can be directed to organelles such as mitochondria via mitochondrial targeting sequences [37]. While targeting signals in protein sequences can be located at the C-terminus and in internal regions, they are most commonly found at the N-terminus [38]. Hence, we expected that a large fraction of mitochondrial proteins in the analyzed mt-cy multigene families would have a mitochondrial N-terminal targeting sequence comparing to their cytosolic counterparts. We used targetP prediction [38] to analyze the presence of N-terminal mitochondrial targeting sequences in human proteins within the mt-cy families (Figure 5A). While over 80% of non-mitochondrial proteins lack a recognizable mitochondrial N-targeting signal, over 50% of mitochondrial proteins have the signal (chi-square p-value is 3.06e-15). This result meets our expectation that the gain or loss of mitochondrial N-terminal sequences plays an important role in directing protein subcellular relocalization after duplication. At the same time, the existence of mitochondrial targeting signals in approximately 20% of non-mitochondrial proteins and our inability to find such a signal in 50% mitochondrial proteins indicates that the N-terminal sequence is not the only modification. Similar sequence modifications might have taken place at C-terminal or internal protein regions that are difficult to identify.

We further investigated the differences in protein functional domains between mitochondrial and non-mitochondrial proteins based on a suggestion that protein function and/or protein functional efficiency can be modified upon the change in its subcellular location [39]. Here we compared the distribution of mitochondrial Pfam domains that were previously found only in eukaryotic (excluding human) mitochondrial proteins [40] among members of mt-cy protein families. We found a significant difference in this distribution (figure 5B): 53% of mitochondrial proteins have mitochondrial domains, but only 16% of non-mitochondrial proteins have such domains (chi-square p-value is 3.5e-9). This result indicates that subcellular relocalizations were characterized by the formation of mitochondrial protein domains or their loss in nuclear copies during evolution.

### Effects of inaccurate or incomplete cellular component annotation

**Figure 6:**
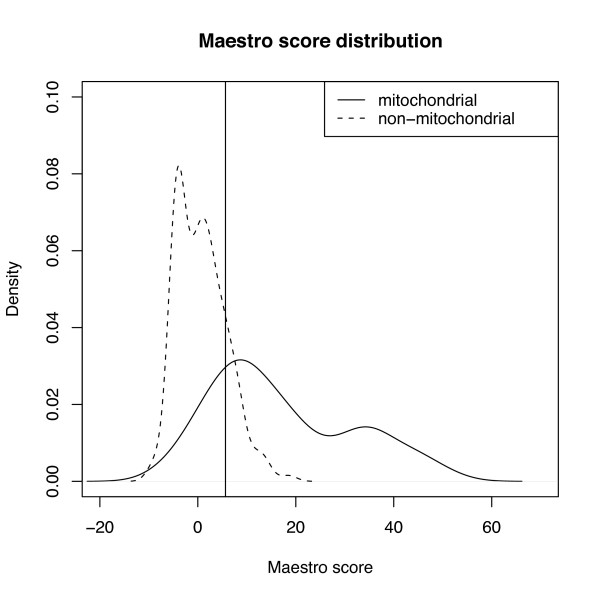
**Maestro score distributions for human mitochondrial and non-mitochondrial proteins**. Maestro scoring system incorporates eight genomic-scale data sets (targeting sequence prediction, protein domain enrichment, presence of *cis*-regulatory motifs, yeast homology, ancestry, tandem-mass spectrometry, coexpression and transcriptional induction during mitochondrial biogenesis) for predicting nuclear encoded mitochondrial proteins [40]. The cutoff score of 5.65 is indicated as the vertical bar.

Knowing accurate protein subcellular localization is important for this study. Although we combined information from several databases to infer protein functional locations, uncertainty still exists in our assignments. To check how these uncertainties would affect our results and conclusions, we reanalyzed all human proteins in this study by applying maestro scores, a scoring system for predicting nuclear encoded mitochondrial proteins in human and mouse that incorporates eight genomic-scale data sets including targeting sequence prediction, protein domain enrichment, presence of *cis*-regulatory motifs, yeast homology, ancestry, tandem-mass spectrometry, coexpression and transcriptional induction during mitochondrial biogenesis [40]. The maestro score distributions of mitochondrial and non-mitochondrial proteins in our dataset are separated with some overlaps (Figure 6). By applying the suggested score cutoff (5.65) to assign subcellular locations to the analyzed proteins [40], we observed 11 out of 59 mt-cy families were grouped into mt-mt category; yet 5 out of 33 mt-mt families were classified to mt-cy families. Even if we do not count the 11 potentially ambiguous families into mt-cy category, still more than 50% of all mitochondrial multigene families have undergone subcellular relocalization after gene duplication.

Limited knowledge of protein subcellular location also prevents us from discovering cases of a different form of subcellular relocalization, called sublocalization, in which duplicated genes become targeted to a subset of their ancestral cellular compartments [21]. For example, the glutamate dehydrogenase family was grouped into mt-mt families since both GLUD1 and GLUD2 are located to mitochondria based on the Genbank and Swissprot annotations. However Rosso et al. recently reported that GLUD1 located to both cytoplasm and mitochondria while GLUD2 became specifically localized to mitochondria owing to a single positively selected amino acid substitution at the N-terminal targeting sequence [21]. In addition, if the ancestral protein had dual localizations but only one of them was annotated, then neolocalization would be inferred instead of sublocalization. The latter problem should be especially pronounced if non-model species are used as outgroups since the localizations of proteins in these species are not thoroughly studied. To investigate the potential effect of this bias, we searched protein localizations in *Drosophila melanogaster *for the families duplicated in the vertebrate lineage. None of these Drosophila proteins are annotated to have dual localizations. Thus available data suggests that neolocalization rather than sublocalization is the prevalent mode of evolution of duplicated genes studied here.

## Conclusion

Protein subcellular relocalization was proposed as an evolutionary mechanism for generating new functional genes after gene duplication [19]. This mechanism was studied in yeast genomes but only received support from individual cases/families in animal genomes [20-22]. Here we systematically investigated human mitochondrial protein families and found that around two thirds of multigene families have protein members that underwent subcellular relocalization after gene duplication. These subcellular relocalizations can occur between mitochondria and another subcellular compartment as well as among several compartments. Comparative sequence analyses showed that the subcellular relocalization processes were primarily enabled via the gain or loss of N-terminal mitochondrial targeting sequences.

After evaluating possible effects of incomplete or incorrect annotations, we conclude that our observation of the subcellular relocalization after gene duplication on the genomic scale was robust to misannotations. Surprisingly our results indicate a scarcity of recent subcellular relocalization events and suggest that protein subcellular relocalization was more important in obtaining new functional genes at the early stages of animal genome evolution. The observation that subcellular relocalization rarely follows recent gene duplications needs to be further investigated because our dataset might be biased in terms of annotation availability. This investigation, together with the previously published finding in yeast [20], indicates that subcellular relocalization is an important mechanism in the retention and gain of function of duplicated genes over the course of eukaryotic genome evolution.

## Methods

### Retrieving human mitochondrial proteins and identifying the paralogs

We used the MitoP2 database http://www.mitop.de:8080/mitop2/ to retrieve human mitochondrial proteins [41]. MitoP2 is a manually annotated database of mitochondrial proteins that integrates computational predictions, proteome mapping, mutant screening, expression profiling, protein-protein interactions and cellular localization. Among 920 inferred human mitochondrial proteins in MitoP2 at the time of our study, we selected 456 that were also annotated as mitochondrial proteins in the Swissprot database. These 456 protein sequences were used to query the Swissprot database using blastp program. The hit sequences with e-value smaller than 0.001 and bit scores equal to or greater than 100 were kept for further analyses. Single linkage clustering was then carried out to cluster the sequences into 305 families [42].

### Ortholog collection and phylogeny construction

Gene families were removed from analysis if all human proteins in the family were products of alternative splicing of the same gene or the records of the proteins no longer existed in Genbank. For each human protein in the remaining 92 multigene families, we retrieved orthologs in mouse (*Mus musculus*), chicken (*Gallus gallus*), fish (*Danio rerio*), fruit fly (*Drosophila melanogaster*), mosquito (*Anopheles gambiae*) and nematode (*Caenorhabditis elegans*) from the Homologene database at NCBI. The subcellular localizations of proteins in each family were inferred from Swissprot and Genbank databases. The protein sequences in each family were aligned with T-coffee using the default settings [43]. The aligned sequences were manually inspected and then imported into the Mega package to construct neighbor joining and parsimony trees with 1000 bootstrap replicates [44]. For each family, we further performed a maximum likehood search with 100 bootstrap replicates as implemented in the PHYML (v.2.4.4) program using the WAG model with estimated amino acid frequencies and 4 gamma categories. The inferred topologies were congruent among different tree-making methods except for some minor differences (not shown). We selected maximum likelihood trees for illustrations.

### Comparative analyses of protein sequences

For human proteins in each family, we retrieved information of N-terminal mitochondrial targeting signal (TARGETP), Pfam domains (MITODOMAIN) and maestro scores developed by Calvo and coauthors for predicting mitochondrial proteins [40]. The distributions of these data were plotted in R package http://www.r-project.org/ for mitochondrial and non-mitochondrial protein members in the analyzed multigene families. The Pearson chi-square tests were applied to test whether the distributional proportions of these genomic criteria were the same for mitochondrial and non-mitochondrial proteins.

## Authors' contributions

XG, DVL and XW designed the study. XW carried out the study and drafted the manuscript. YH participated in the design of the study and helped in script writing for acquisition of data. XG and DVL supervised the study and participated in writing the manuscript. All authors read and approved the final manuscript.
